# 

**Published:** 1891-02-14

**Authors:** 


### NOTICE TO CORRESPONDENTS.

All MS., Letters, Books for Review, and other matters intended for the Editor should be addressed The Editor, The Lodge, Porchester Square, London, W.

The Editor cannot undertake to return rejected M.S., even when accompanied by stamped directed envelope.

### Notice to Advertisers and Subscribers.

All Advertisements, Orders for Copies of The Hospital, and Business Communications should be addressed to The Publisher, *not to the Editor*, The Hospital, 140, Strand, W.C.

The Cost of Subscription, post free, is as follows:—Per week, 1½d.; per quarter, 1s. 8d.; per half-year, 3s. 3d.; per annum, 6s. 6d. Foreign:—Per annum, 8s. 8d.; payable, in all cases, in advance.

### Scraps and Gleanings.

January 25th was Hospital Sunday in Nottingham and Notts.

A new ward has been opened in the Bath Royal United Hospital.

Mr. Ludwig Mond has given £100 and E. A. H. £500 to the Hospital Sunday Fund.

The buildings of the Torbay Hospital have been found to be insanitary. A new hospital will be erected.

Mr. Henry Andrew has been appointed Assistant House Surgeon to the Devon and Exeter Hospital.

The total number of persons treated at Halifax Infirmary last year was 6,247, as against 5,984 in 1889.

During the month of January, 529 patients were under treatment at the Royal Ear Hospital, Frith-street.

The Court of Common Council have voted a gratuity of £210 to the North-Eastern Hospital for Children.

The collections in the churches and chapels in Sheffield on Hospital Sunday amounted together to £2,197 5s. 9d.

The London Hospital has received from the Queen a further gift of twenty pheasants for the use of the patients.

The hospital at Skopen, Odessa, has been entirely destroyed by fire. Fourteen of the patients were burned to death.

Aberdeen Royal Infirmary has been enlarged by the addition of surgical, clinical, and administrative departments.

A sum of nearly £80 has been collected for the General Hospital, Nottingham, by means of weekly subscriptions of one penny.

Professor von Mosetig, chief of the surgical ward at the Wieden Hospital, Vienna, believes that he has discovered a cure for cancer.

Mr. R. E. Crosse has been elected to the post of House Surgeon in the Norfolk and Norwich Hospital, in the place of Mr. Nanee, who has resigned.

North-Eastern Hospital for Children, Hackney Road.—The Corporation of the City of London have granted a donation of £210 to this hospital.

The total receipts of the Bradford Joint Hospital Fund during 1890 was £5,321 13s. 9d., being the largest revenue of any year since the joint fund was established.

Sir Thomas Sutherland, K.C.M.G., M.P., has consented to preside at the annual court of the Seamen's Hospital Society, to be held on Tuesday, February 17th.

The Lord Mayor has consented to preside at a festival dinner in aid of the Royal National Hospital for Consumption, Ventnor, to be held at the Hotel Metropole on April 13th.

At the Teignmouth, Dawlish, and Newton Infirmary and Dispensary, 245 in-patients and 1,331 out-patients have been under treatment during 1890. The receipts amounted to £953 12s. 2½d., and the expenditure to £1,057 17s. 6½d.

Owing to the increase in the number of applications to the Children's Hospital, Cold Ash, Newbury, it will be necessary to enlarge the hospital. A site for a new wing has been given, and £300 has been collected towards the cost of erecting and maintaining it.

The Local Government Board have, in pursuance of the powers vested in them under the Public Health Act, 1875, issued a provisional order for the formation of a Joint Hospital Board for the districts of Edmonton, Enfield, South Hornsey, and Tottenham.

Sir William Gull has founded a Gull Studentship in Pathology at the Medical School of Guy's Hospital, of the yearly value of £125, with a further allowance of £25 for apparatus. It will be tenable for three years, and may be renewed for two years more.

The annual meeting of the Royal Hospital for Children and Women, Waterloo Bridge Road, is fixed for Friday, March 6th next, at 6.30, at the Mansion House. The Lord Mayor will preside, supported by the Lady Mayoress, the Sheriffs of London, and their ladies. A crowded meeting is expected.

The Duke of Clarence and Avondale will preside, on May 4th next, at the festival dinner, at the Hotel Métropole, in aid of the Royal Hospital for Children and Women, Waterloo Bridge Road. The Secretary (Mr. R. G. Kestin) will be pleased to receive the names of gentlemen willing to act as stewards on the occasion.

During the past year the Dowager Lady Howard de Walden has made a munificent gift to the West Kent Hospital. The whole of the top floor has been fitted by her as a women's ward for eight free beds. A ward for special cases and bed-rooms for the staff have also been provided, and Lady de Walden has placed in the hands of the trustees ample funds for their maintenance.

The Mayor of Liverpool presided at the annual meeting of the friends and subscribers of the Northern Hospital. From the report, which was adopted, it appeared that the sum of £1,484 was received from the Hospital Saturday and Sunday Fund. There had been 1,947 in-patients, and 5,440 out-patients. The income from all sources amounted to £8,302 19s. 8½d., and the total expenditure was £6,841 17s. 5d., leaving a balance of £1,461 2s. 3½d.

The annual meeting in connection with the Weymouth Royal Hospital was held on January 15th under the presidency of the Mayor. The medical report showed that there had been 91 in-patients and 1,454 out-patients under treatment during 1890. From the balance-sheet it appeared that the expenditure had amounted, together with an adverse balance from 1889 of £40 17s. 10d., to £609 8s. 3d. The income was £609 18s., leaving a balance in hand of 9s. 9d. The report was adopted.

The annual meeting of the Governors and friends of the Children's Hospital, Birmingham, was held in the Medical Institute. Mr. C. A. Smith-Ryland presided. The report showed that although every possible economy had been practised by the Committee of Management, the expenditure exceeded the income. Early in the year an anonymous friend gave £1,000 for the endowment of a cot for chronic cases, and later in the year a second generous offer came from Mr. R. Cadbury, who proposed to purchase Mosely Hall for a convalescent home for children, which offer had been gratefully accepted.

### General Charities.

[This Column is devoted to an account of work of charitable institutions other than Medical Charities. The Editor will be glad to receive reports or news of work at 140, Strand. *Philanthropy* should be plainly written on the envelope.]

The demands on the various clerical societies have, during recent years, undoubtedly been heavy, and yearly it seems to become more difficult to cope with the ever-present poverty of the majority of clergy, many of whom are in receipt of incomes utterly inadequate for any provision for their widows and children whom they leave behind them. The operations of the **Corporation of the Friend of the Clergy** are divided into two branches, the principal one being devoted to the granting of pensions to the widows and orphan unmarried daughters of the clergy. Widows who are elected at 50 years of age receive pensions of £30, those at 60 years £35, and those at 70 years or over £40. Two elections take place yearly, in May and November, and on the average about ten pensioners are elected during the year. The second branch is confined to affording temporary assistance to clergymen and their families in times of pressing need and difficulty, and it is usual to distribute about £300 every January for this purpose. In addition to donations the committee are always glad to receive any articles of clothing for distribution among some of the larger families of the poorer clergy, addressed to the Secretary, Rev. Henry Jona, 27, Bedford Street, Strand, W.C.

**The Army and Navy Pensioners' Employment Society**, which has branches in Dublin, Glasgow, and Manchester, has a claim upon the public generally, who are benefited by having good servants supplied to them, as well as upon the service in particular. We trust, therefore, that the fear was unfounded which Lieut.-General W. Drysdale expressed at the beginning of this year that unless the society received very substantial support it would not only be impossible to meet current expenses without selling out capital, but it would be necessary to reduce one of the branches. Judging by the income of the society, which is under £900 a year, the support which the army or the general public gives to it is not rashly extravagant. We do not see how the expenditure could well be smaller, and at the same time efficient. All men discharged from the army or navy with pensions are eligible to be registered for employment through the aid of the society. All that is required is the production of the certificate of discharge and pension certificate, and satisfactory proof of good character. When more than six months have elapsed between the date of his discharge and his application for registration by the society satisfactory testimonials must also be produced of his conduct during the interval. When once a pensioner's name has been registered, misconduct only will deprive him of the power of applying to the society for employment at any time. Secretary, Lieut.-Colonel E. P. Newman, 20, Charing Cross, S.W.

### The Student of Medicine.

[All communications for this Supplement should be addressed to The Editor of The Hospital, at 140, Strand, with the words, "Students' Column," written in the left hand top corner of the envelope.]

### EDITORIAL NOTES.

Inter-Hospital Rugby Challenge Cup.—The first round of the Inter-Hospital Rugby Cup competition came to a conclusion on February 2nd, and the winners have now entered on the second stage of the contest. The fact that there had been a total absence of football from the metropolis for two months or more, was naturally expected to lower the standard of play, and consequently to introduce a larger element of chance than would otherwise have existed. As a fact, however, the play throughout, and especially in the Thomas's v. Mary's, and Middlesex v. Bart.'s matches, was well up to the best hospital standard. A noticeable feature in all the matches was the superiority of the forward over the back divisions; nearly all the hospitals putting fairly strong forwards on the field, whilst Thomas's and Guy's alone were really strong behind, Middlesex and George's being the pick of the rest. The best game was that between Bart.'s and Middlesex, the forwards being very evenly matched, but the latter, without one of the best three-quarters, Lee, were decidedly stronger behind. The superiority of the back division also secured Guy's and George's the victory over London and University, in both of which cases the forward play was of a fairly even character; and even the redoubtable Thomas's forwards by no means had entirely their own way against Mary's. In the second round Thomas should run up a big score against George's, and Guy's should easily dispose of Charing Cross; the first-named should apparently win the final without much trouble, whilst Guy's and Middlesex, if they be drawn together in the semi-final, should make a close fight for second honours, although the former are generally considered the stronger team.

### NOTES FROM THE SCHOOLS.

### CAMBRIDGE UNIVERSITY.

Sir G. M. Humphrey, Professor of Surgery, has been appointed a member of the General Board of Studies.

Joseph Griffiths, M.A., M.D., Edin. assistant to the Professor of Surgery, has been nominated an additional member of the Special Board for Medicine.

Degrees.—The following degrees were conferred at the congregation on Thursday, Jan. 15th: M.B. and B.C.—Walter Winslow, B.A., Caius (thesis, the Cause of Death in Enterica); Robert Stanley Thomas, B.A., Jesus (thesis, Typhlitis); Jermyn Francis Gillett, B.A., Sidney (thesis, Treatment of Pneumonia by the Ice-bag); William Hubert Sale Fosbery, B.A. (thesis, Adhesion of Ovarian Cysts).

At Sidney Sussex College an exhibition of £30 in natural science has been awarded to E. M Corner, of Epsom College.

### STUDENTS' MEDICAL SOCIETIES.

### Middlesex Hospital.

A meeting of this society was held on Thursday evening, January 15th, Mr. A. Pearce Gould in the chair. Mr. Kenneth Lawson read a paper on the "History of Anatomy," in which he carefully traced its development from earliest Egyptian times up to the present day. Briefly reviewing the men who had distinguished themselves in anatomical research, he referred to Aristotle as having made a classification of animals, and to Pythagoras as having recognized the distinction between arteries and veins, though the former scientist considered the brain as separated entirely from all the organs of sense, whilst the latter embraced the then popular theory of the vessels being filled with air. Mr. Lawson showed how the science made rapid strides after the time of Alexander, when Herophilus and others carried on their dissections, how, for some 200 years following, the study advanced but little, until Galen revived it, and added greatly to the facts already ascertained. Harvey and Morgani were, in their turn, discussed, and an interesting account was given of John Hunter's coming to London, and joining the school already founded by his brother William, of the discoveries in anatomy which resulted, and of the founding of the College of Surgeons. In conclusion, Mr. Lawson referred to the difficulties which those desirous of studying anatomy underwent, both from the paucity

The Hospital Advertiser.

### THE STUDENT OF MEDICINE—(*continued*).

of dissection subjects, and from the opposition of those who objected to the practice of human dissections at all, and showed how these difficulties drove men the more to the study of comparative anatomy—a study which, though of great importance, cannot be undertaken by the present generation of students in a four years' course. The President referred to John Hunter as being the greatest of all anatomists from the vast range of his study, and in drawing a comparison between the two brothers, he thought it a mistake to attribute most knowledge and scientific research to William Hunter on account of his better education and ability in writing. Mr. Gould thought an increase in study of comparative anatomy advisable, and that if it were adopted, the paucity of dissecting-room subjects would cease. Mr. W. A. Ward, agreeing with most that had been said, was anxious to know where the fresh ground for anatomical research in the future lay. The next meeting was held on Thursday evening, January 29th, when Mr. T. Carwardine read a paper entitled "Phrenology and the Localization of Function." Histological and pathological specimens illustrating blood vessels, lymphatics and ductless glands were shown.

#### Westminster Hospital Guthrie Society.

The monthly meeting of this society took place on January 8th, at eight o'clock. Mr. Charles Stonham was in the chair. The minutes of the last meeting having been read and signed, Mr. H. Betts, house physician, was elected to the office of Vice-President, *vice* A. W. Harrison resigned, Mr. Sharman was elected Treasurer, *vice* H. Betts, resigned. Mr. W. S. Spencer, assistant surgeon to the hospital, read a paper on "Some objections to Darwinism." Some discussion followed, the opinions of the speakers being decidedly in favour of the Darwinian hypothesis. There was subsequently a microscopical demonstration of pathological specimens. The meeting did not terminate till half-past ten, the paper having been of a somewhat lengthy character.

#### APPOINTMENTS.

At the Tetbury Cottage Hospital, G. W. W. Ashdown, M.D., M.B.Edin., M.R.C.S., has been appointed medical officer. At the Southport Hydropathic Hospital, Henry Blumberg, M.D. Prague, L.R.C.P.Lond., has been appointed honorary medical officer. At the Warwick Provident Dispensary and Cottage Hospital, T. W. Bullock, M.R.C.S., has been reappointed senior surgeon. At the Ripon Cottage Hospital and Dispensary, H. J. Daggett, M.B., B.C. Camb., L.R.C.P. Lond., M.R.C.S., has been appointed house surgeon. At the Cumberland Infirmary, Carlisle, C. H. Powers, L.R.C.P., M.R.C.S., has been appointed house surgeon. At Smedley's Hydropathic Establishment, Marlock Bridge, William C. Sharpe, M.B., C.M.Edin., has been appointed resident junior physician. At the Royal Albert Hospital, Devonport, L. S. Senhouse, M.B., C.M. Edin., has been appointed assistant house surgeon. At the West of England Hydropathic Establishment, Limpley Stoke, Bath, Charles J. Whitby, B.A., M.B. Camb., has been appointed resident physician.

#### VACANCIES.

The following vacancies are announced this week, the amount of salary in each case being given after the name of the institution :

**Resident Medical Officers.**

Addenbrooke's Hospital, Cambridge, Resident House Physician—Salary £65 per annum, with Board, &c.

Brixton, Streatham, and Herne Hill Dispensary, House Surgeon—Salary £150, &c.

Central London Sick Asylum District, Assistant Medical Officer and Dispenser—Salary £100, with Board, &c.

City of London Hospital for Diseases of the Chest, Victoria Park, E., House Physician for six months, board, &c.

Chichester Infirmary, Chichester, House Surgeon—Salary £80, with Board, &c.

Doncaster General Infirmary and Dispensary. House Surgeon—Salary £100, with board, &c.

County Donegal Infirmary, House Surgeon and Registrar—Salary £60. &c.

Dorset County Hospital, Dorchester, House Surgeon—Salary £70, with Board, &c.

East Suffolk and Ipswich Hospital, Assistant House Surgeon—Salary £20, with Board, &c.

Hospital for Consumption and Diseases of the Chest, Brompton, House Physicians—Board, &c.

Hospital for Sick Children, Great Ormond Street, Bloomsbury, W.C., House Physician for one year—Salary £60, with Board, &c.

Liverpool Infirmary for Children, Assistant House Surgeon for six months—Board, &c.

National Dental Hospital, 149, Great Portland Street, W., House Surgeon—Salary £50.

THE STUDENT OF MEDICINE—(*continued*).

Newcastle-on-Tyne Dispensary, Resident Medical Officer—Salary £250, &c.
Nottingham General Hospital, Resident Surgical and Resident Medical Assistants for six months—Board, &c.
National Sanatorium for Consumption and Diseases of the Chest, Bournemouth, Clinical Clerk for four months—Board, &c.
Scarborough Hospital and Dispensary, Scarborough, House Surgeon for twelve months—Salary £80, with board, &c.
Southern Hospital, Clifford Street, Manchester, House Surgeon.

**Non-Resident Medical Officers.**

St. George and St. James's Dispensary, Surgeon.
Brown Animal Sanatory Institution, Professor Superintendent. Salary £250.
County Council for the County of Derby, Medical Officer of Health. Salary £600 per annum, with £200 extra for travelling and other expenses.
County of Lanark, Medical Officer for the county. Salary £700, &c.
Farringdon General Dispensary and Lying-in Charity, 17, Bartlett's Buildings, Holborn, E.C., Honorary Physician Accoucheur.
London Fever Hospital, Liverpool Road, N., Physician and Assistant Physician.
Owens College, Manchester, Demonstrator of Pathology. £140 per annum.
Queen's College, Cork, Lecturers in the following subjects—Psychological Medicine, Pathology, Ophthalmology, Hygiene, Otology.
Royal Academy of Arts, Professor of Anatomy.
St. Mark's Hospital for Fistula, etc., City Road, E.C., Anæsthetist. Honorarium, £50 per annum.
University of Glasgow, Examiners in Medicine.

### ATHLETICS.

### Football.

Association Rules.—January 31.

*Guy's Hospital* were beaten by *Marlow*, at Marlow, by two goals to nil. *Oxford University* beat *Crusaders*, at Oxford, by five goals to one. *St. Bartholomew's Hospital* beat *Old St. Marks*, at Barnes by six goals to one.

*Great Marlow v. Guy's Hospital.*—This match was played on the Crown Meadow, Marlow. Lunnon, losing the toss, started the ball soon after three o'clock. During the first half of the game the play was pretty even. The home team showed very inferior form. Fifteen minutes from the kick off the captain of the Marlow eleven, R. A. Lunnon, sustained a wound on his forehead and had to leave the field, but after a quarter of an hour returned, amid cheers, with his head bandaged. Half-time arrived without a goal being obtained. On change of ends, however, three goals were obtained in four minutes, the first by Pendried for the medicals, and the second by F. S. Lunnon by a long well-directed shot. Just afterwards Pendried lost a good chance to equalise when the Marlow goal was at his mercy. Failing to score, Guy's were defeated by two goals to one.

*St. Bartholomew's Hospital v. Old St. Marks.*—The tie between these clubs were decided at the Limes, Barnes, on Saturday, and resulted in the hospital being victorious by six goals to one. St. Bartholomew's started against the wind, and the ball was rushed through after about ten minutes' play. Fast play followed, and just before half-time Dixon registered a second goal for the visitors. After crossing over Wilson kicked the third and Fernice the fourth goal. The Old Boys then retaliated, and from a pass from Schumacher, Redmond obtained their first and only goal. Hogarth then scored twice in quick succession, and resulted as above stated.

Rugby.

Inter-Hospital Challenge Cup.—The results of the first round are as under: January 26, *St. Thomas's* beat *St. Mary's* by four goals and one try to one try. January 27, *Middlesex* beat *St. Bartholomew's* by one goal to nil. January 29, *St. George's* beat *University* by two goals and one try to nil. January 30, *Charing Cross* beat *Westminster* by two goals and two tries to one goal. February 2, *Guy's* beat *London* by four goals and two tries to one goal. The two ties in the second round will be played on February 10 and 12, the semi-final on February 24, and the final on March 5. The rules of scoring have been altered to the following: A try, or penalty goal, one point; a goal from the field of play, two points; a goal from a try, three points.

January 31st.

*Cambridge University* beat *Blackheath* at Blackheath, by three goals two tries to two tries. *St. Thomas's Hospital* was beaten by *Harlequins* by one try to nil. *London Hospital* beat *Saracens*, at Walthamstow, by one try to nil. *Oxford University* beat *Kensington*, at Oxford, by three goals and two tries to nil. *St. Bartholomew's Hospital* beat *Old Merchant Taylors*, at Balham, by two goals to nil. *London Hospital* were beaten by *Saracens*, at Walthamstow, by one goal to nil.

February 4th.

*Edinburgh University* (scratch) beat *Fetter College* by four goals and four tries to nil.

**EVENTS FOR THE MONTH OF FEBRUARY.**

[Items intended for this column must reach the office, 140, Strand, W.C., not later than first post on Monday mornings.]

For list of Students' Medical Societies' Meetings and Football Matches during February, see The Hospital for Feb. 7th.

